# The smallest chemical reaction system with bistability

**DOI:** 10.1186/1752-0509-3-90

**Published:** 2009-09-08

**Authors:** Thomas Wilhelm

**Affiliations:** 1 Theoretical Systems Biology, Institute of Food Research, Norwich Research Park, Colney Lane, Norwich NR4 7UA, UK

## Abstract

**Background:**

Bistability underlies basic biological phenomena, such as cell division, differentiation, cancer onset, and apoptosis. So far biologists identified two necessary conditions for bistability: positive feedback and ultrasensitivity.

**Results:**

Biological systems are based upon elementary mono- and bimolecular chemical reactions. In order to definitely clarify all necessary conditions for bistability we here present the corresponding minimal system. According to our definition, it contains the minimal number of (i) reactants, (ii) reactions, and (iii) terms in the corresponding ordinary differential equations (decreasing importance from i-iii). The minimal bistable system contains two reactants and four irreversible reactions (three bimolecular, one monomolecular).

We discuss the roles of the reactions with respect to the necessary conditions for bistability: two reactions comprise the positive feedback loop, a third reaction filters out small stimuli thus enabling a stable 'off' state, and the fourth reaction prevents explosions. We argue that prevention of explosion is a third general necessary condition for bistability, which is so far lacking discussion in the literature.

Moreover, in addition to proving that in two-component systems three steady states are necessary for bistability (five for tristability, etc.), we also present a simple general method to design such systems: one just needs one production and three different degradation mechanisms (one production, five degradations for tristability, etc.). This helps modelling multistable systems and it is important for corresponding synthetic biology projects.

**Conclusion:**

The presented minimal bistable system finally clarifies the often discussed question for the necessary conditions for bistability. The three necessary conditions are: positive feedback, a mechanism to filter out small stimuli and a mechanism to prevent explosions. This is important for modelling bistability with simple systems and for synthetically designing new bistable systems. Our simple model system is also well suited for corresponding teaching purposes.

## Background

Bistability is key for understanding basic phenomena of cellular functioning, such as decision-making processes in cell cycle progression, cell differentiation, and apoptosis [1]. It is also involved in loss of cellular homeostasis associated with early events in cancer onset [2] and in prion diseases [3]. A recent review discussed different bistability phenomena in bacteria, such as different phenotypes in clonal populations being important for the origin of new species [4].

Bistable switches are typically enabled by positive feedback loops in signal transduction networks. Here a sufficiently strong (external) signal switches on a self-amplifying process leading to expression of the corresponding target genes. Due to the corresponding hysteresis effect this process can retain its activity without a persistent signal. Such switches are therefore called 'decision-making'. One often discussed example is the restriction point control for the regulation of G1-S transition of the mammalian cell cycle, where recently a detailed small ordinary differential equation (ODE) model was presented [5]. The G2-M transition was also described as a toggle-switch [6]. Oocyte maturation is an example for the involvement of a bistable system in cell differentiation [7,8]. Biochemical switches have also been found in nutrient utilization in bacteria [9], mating response in yeast [10], and synaptic memory processing [11]. Interestingly, just quite small parts of the large signal transduction systems (for instance a single layer of a MAPK cascade) can already induce bistability. This was demonstrated for the epidermal growth factor receptor system [12] and other kinase phosphatase systems [13,14].

Given its outstanding biological importance, it is clear that bistable switches also attracted the attention of theoretical biologists. A frequently discussed problem is the necessary (and/or sufficient) condition for bistability. The central result goes back to the work of Clarke [15] and Thomas [16]: autonomous differential systems can possess multiple steady states only under the presence of positive feedback loops [17,18]. It was also argued that the feedback needs 'some type of non-linearity' or 'ultrasensitivity' for inducing bistability [19]. Different types of ultrasensitivity have been discussed [20], but formulated in the most general manner that means the system needs some mechanism for filtering out small stimuli to enable a stable 'off' state [20-22]. Feinberg's chemical reaction network theory (CRNT) even gives necessary and sufficient conditions for bistability, by restricting to special mass-action kinetic (MAK) systems [23]. In a recent application example a single layer of a MAPK cascade was studied and the region in parameter space being relevant for bistability was analytically described [24]. However, for larger systems CRNT leads to cumbersome calculations, but the applicability could recently be improved by just studying important subnetworks [25] which are based on the concept of elementary flux modes [26].

Another approach for identifying necessary structural conditions for any dynamic behaviour is the identification of the corresponding minimal systems. Such systems have the advantage of being "simple enough to understand at an intuitive level" [19] and are well suited for different basic studies. For instance, the Lotka-Volterra system [27,28], the Higgins-Selkov-oscillator [29-31], and the "Brusselator" [32] have been studied extensively. Some years ago we identified the smallest chemical system with Hopf bifurcation [33]. Minimal MAK systems are summarized in Table 1. Recently, different 'smallest' or 'minimal' bistable systems for cell polarity [34] and G protein signalling [35] have been presented, as well as 'the smallest multistationary mass-preserving chemical reaction network' [36]. However, these systems are still too large to represent a minimal bistable system according to definition (1). Many different reaction topologies with 3 and 4 molecules have also been analysed computationally for the possibility of bistability [37]. Although this type of bistability detection may miss some bistable systems, the authors found nevertheless many topologies with switching behaviour (10% of tested configurations). The identified 'minimal' system contained 3 variables (5 reactants, 2 conservation relations) and 6 reactions. The bistable one-dimensional Schloegl system [38] contains trimolecular reactions and can therefore not represent a realistic elementary chemical system. Elementary chemical reactions are at most bimolecular.

**Table 1:** Distinguished minimal MAK systems

**System**	**Reaction scheme**	**MAK model (ODEs)**	**Ref.**
**Minimal bistable MAK system**			[38]

**Minimal bistable chemical system**			This paper

**Minimal oscillating MAK system**			[27,28]

**Minimal MAK system with limit cycle**			[29-31]

**Minimal chemical system with limit cycle **			[33]

*S *and *P *denote constant substrates and products.

Here we present and discuss the smallest bistable chemical reaction system. Application of our previously presented Instability Causing Structure Analysis (ICSA [39]) leads to additional insight into system functioning.

## Results

### The smallest bistable chemical reaction system

We define the smallest chemical system (contains only mono- and bimolecular reactions, reversible reactions are considered as two irreversible ones) by the following criteria in decreasing order of importance:

(1)

According to this definition, the following bistable system is unique (Methods section contains the proof for this statement).

(2a)

Assuming spatially homogeneous conditions, the system can be described by the two-component mass-action kinetic ODE system (*S *is incorporated into *k*_1_):

(2b)

Due to its simplicity, the mathematical analysis of the system is simple as well. The system has two elementary flux modes [26], following directly from the two nullspace vectors of the corresponding stoichiometric matrix

(2c)

One mode uses reactions 1-3, and the other reactions 1,2, and 4. Bistability can, of course, only arise if all reactions are active. Introducing dimensionless quantities (*x*/*c *→ *x*, *y*/*c *→ *y*, *k*_1_/(*k*_2_*c*) → *k*_1_, *k*_3_/*k*_2 _→ *k*_3_, *k*_4_/(*k*_2_*c*) → *k*_4_, *tk*_2_*c *→ *t*), we set *k*_2 _= 1, without restriction of generality (*k*_1_, *k*_3_, *k*_4 _> 0). The system has three steady states: , , with the discriminant *D *= *k*_1 _- 4*k*_3_*k*_4_. A saddle-node bifurcation occurs at *D *= 0, the three steady states are real if *D *> 0. The second and third steady state is always positive.

Generally, in two-component systems a steady state is locally stable if the trace *tr *and determinant *det *of the Jacobian at this point are negative and positive, respectively (node if 4*det *<*tr*^2^, focus otherwise). If the corresponding determinant is negative, the steady state is a saddle point. It can be seen from the Jacobian  that its trace is always negative (phase flow of system (2) is confined to the positive part of the phase space). This excludes Hopf bifurcations (arising at *tr *= 0) and it means the system is dissipative, i.e. phase-space contracting all over the phase space (trace = two-dimensional Lyapunov exponent). The determinants of the Jacobian at the three steady states read *k*_1_*k*_4_, , and , respectively. Therefore, the first and third steady state are always locally stable, the second is always locally unstable, a saddle-point (determinant of Jacobian at second steady state always negative, cf. point 3 in Methods). Simple calculation shows further that 4*det *<*tr*^2 ^at the first and third steady state, so these are always stable nodes.

**Figure 1:**
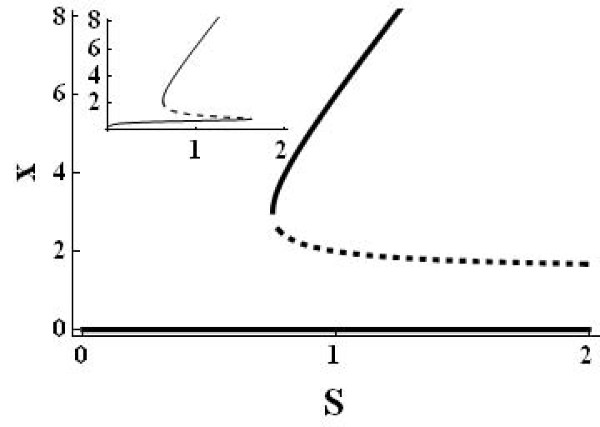
**Signal-response curve (bifurcation diagram) of system (2) for the parameters ***k*_1 _= 8, *k*_2 _= 1, *k*_3 _= 1, *k*_4 _= 1.5. Solid lines indicate locally stable steady states, the dashed line locally unstable steady states. The inset shows the signal-response curve if an additional small constant influx into X (here 0.6) is assumed (enabling a positive 'off' state, leaving the 'on' state and bifurcation point nearly unchanged). This is the classical toggle switch (terminology of Tyson et al. (6), others use the term toggle switch to describe a double negative (i.e. positive) feedback loop (4)) picture enabling the hysteresis cycle: starting with low values and increasing the signal continuously increases the response, until the saddle-node bifurcation at about S = 1.7 is reached. Further increase of the signal leads to a sudden jump of the response to the upper steady state. Decreasing the signal now leads to a continuous decrease of the response, the systems stays in the upper steady state until the left bifurcation point is reached where the response jumps back to the lower steady state.

For *k*_1 _= 8, *k*_3 _= 1, *k*_4 _= 1.5 the second and third steady state are  and , respectively. Figure 1 shows a corresponding signal-response curve, also called bifurcation diagram [6]. The signal is the concentration of the constant outer substance S (assuming for the bimolecular rate constant *k*_*bi *_= 1, the concentration S is identical to the apparent rate constant *k*_1 _= *k*_*bi*_*S*), the response is the steady state concentration of an internal reactant, here X. The saddle-node bifurcation occurs at *S *= 3/4. Beyond that point the system has two stable steady states. It follows from  that  (for fixed other parameters), so the real dynamic behaviour of the system is that of a toggle-switch (Figure 1): for sufficiently large *k*_1 _small fluctuations in the concentrations would drive the system to the positive steady state (the 'on' state).

**Figure 2:**
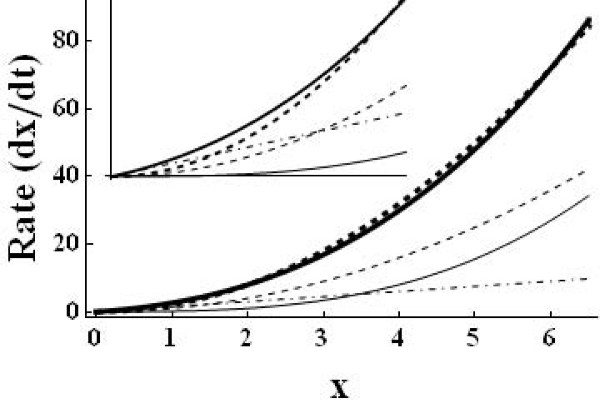
**Rate curves **[6]** of system (2) for the parameters ***k*_1 _= 8, *k*_2 _= 1, *k*_3 _= 1, *k*_4 _= 1.5. The thick solid line is the rate of the removal of reactant X (sum of the negative terms in ) and the thick dashed line the rate of production (positive term in ). The three crossings indicate the three steady states . The thin lines show the contributions of the three degradation terms separately: quadratic term *k*_2_*x*^2 ^dashed, the effectively cubic term *k*_3 _*xy *solid, and the linear term *k*_4 _*x *dotdashed. The inset shows a zoomed version for *x *< 2.1.

Figure 2 shows rate curves of system (2). It can be seen that the three crossings of production and degradation rate (i.e. the three steady states) are due to the different contributions of the three degradation terms. This implies a simple general procedure for designing bi- or multistable systems: a bistable system can be created with one function for production and three different functions for degradation, e.g. a linear, a quadratic, and a cubic one as in our simple example system. Accordingly, tristable systems require 5 different functions to enable 5 crossings (three stable and two unstable steady states, cf. point 3 in Methods), and so forth for more steady states. This observation helps constructing minimal and/or realistic models of more complicated multistable systems. It can also be a starting point for the design of real bistable systems, for instance in synthetic biology. Note that all enzyme kinetic rate laws can be modelled with polynomial ODEs [40].

### The Instability Causing Structure Analysis (ICSA) of system (2)

Recently we presented a new method for topological network analysis of dynamical systems, the *Instability Causing Structure Analysis *(ICSA [39]). Standard stoichiometric network analyses (such as elementary flux mode calculations [26]) are based on the assumption of steady states and lead to linear constraints in flux space. ICSA is a nonlinear network analysis. It is based on the assumption of locally stable steady states. The additional demand for local stability yields additional nonlinear constraints for the steady state flux space [39].

ICSA needs no knowledge about kinetic details. It can be applied to (bio)chemical systems where just the stoichiometric matrix is known (or even just the signs of its elements) or to signal transduction/gene regulatory networks represented by interaction graphs [18] (also called incidence graph [21] or causal influence graph [41]). ICSA leads to additional insight into system functioning by identifying all contained feedback loops and all the corresponding instability causing structures (ICS). An ICS is either a single feedback loop or a special combination of feedback loops [39]. ICSA yields a necessary condition for local instability of any steady state of the system: if there is no ICS all potential steady states are locally stable. For two-dimensional systems this also implies that the system has just one steady state (cf. point 3 in Methods).

**Figure 3:**
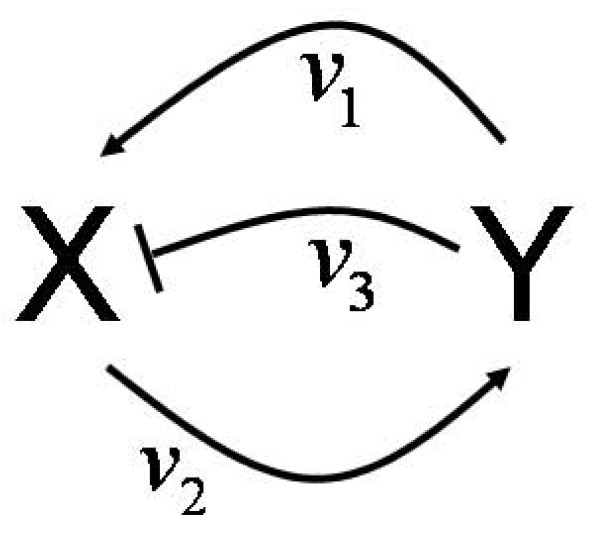
**Interaction graph of system (2)**. It follows directly from the off-diagonal elements of the general Jacobian (3). The positive feedback loop is the only instability causing structure (ICS) in the system, allowing for a locally unstable steady state (presupposition for bistability).

We apply ICSA for additional analysis of system (2). The stoichiometric matrix **S **is given in (2c). Multiplication of **S **with the reaction velocity substrates ector (contains the substrates for each reaction) (*v*_1 _(*y*) *v*_2 _(*x*) *v*_3 _(*x*, *y*) *v*_4 _(*x*))^*T *^leads to . Differentiation yields the general Jacobian **J**_G _(39) of system (2):

(3)

where the indices x and y denote the corresponding partial differentiation. The off-diagonal elements represent the fundamental activating and inhibiting interactions in the system: the positive *v*_2*x *_in **J**_**G**21 _shows that x activates y by the second reaction, and equivalently for the two terms in **J**_**G**12 _: *v*_1*y *_→ y activates x by the first reaction, - *v*_3*y *_→ y inhibits x by the third reaction. Figure 3 shows the corresponding interaction (incidence) graph summarizing these interactions. Interaction graphs can often be found in the biological literature and corresponding databases (KEGG [42]; BIOBASE [43,44]; Dynamic Signaling Maps . ICSA [39] was developed for structural analyses of (bio)chemical systems (KEGG [42]; BRENDA [45]) AND such interaction graphs.

The system contains one positive and one negative feedback loop. The positive loop is the necessary structural condition for bistability [18]. The second often discussed ingredient for bistability is any mechanism for filtering out small stimuli to make the off-state stable. Ferrell and Xiong [20] discussed different such mechanisms, as ultrasensitivity and back reaction saturation. Our system has the simplest mechanism to stabilize the off-state: the monomolecular efflux reaction 4. Analysis shows that without this reaction the second (unstable) steady state merges with the zero off-state making it (weakly) unstable. In fact, Figure 2 (especially the inset) shows that it is the linear degradation term that makes the overall degradation rate higher then production for low X concentrations.

System (2) is the minimal chemical reaction system with bistability. Therefore, any ingredient is essential. That also means, without the negative loop (without reaction 3 being catalyzed by Y) this system cannot be bistable. In fact, the negative feedback prevents explosion of the system: without reaction 3 the system has just one locally stable off-state and one unstable positive steady state. Figure 2 shows that without the cubic term there are just two crossings of the rate curves.

Summarizing, system (2) contains the three different necessary conditions for bistability: (i) a positive feedback loop, (ii) a mechanism for filtering out small stimuli, and (iii) a mechanism for preventing explosion. Interestingly, the third condition lacks discussion in the literature [19,20,22]. Moreover, these three conditions can be related to the three degradation terms in the balance equation of X and so to the last three of the four reactions in (2). The first reaction represents the production of the system, it is the only input. The second reaction closes the positive feedback cycle. Reactions three and four are degradation reactions. The third is the main degradation for higher concentrations, it prevents explosion. The fourth reaction filters out the small stimuli.

The first step in the general ICSA procedure is the analysis of feedback cycles resulting from the off-diagonal terms of the general Jacobian. The second step is the identification of the topological structures that actually cause instability. As mentioned above, standard local stability analysis of two-component systems needs consideration of the Jacobian's trace and determinant *det*. In ICSA we study the general Jacobian. It follows from (3) that the trace is always negative and *det *= *v*_2*x *_*v*_3*y *_+ *v*_1*y *_(*v*_3*x *_+ *v*_4*x *_- *v*_2*x*_). The only negative term in *det *comprises reactions 1 and 2, so this positive feedback cycle is the only ICS of the system. The negative feedback cycle cannot cause any instability here.

## Discussion

The minimal bistable chemical reaction system (2) is based on definition (1). This definition is based on a chemical/physical point of view, but other definitions might also be possible. The definition for the smallest chemical reaction system with Hopf bifurcation [33], for instance, was more mathematically motivated: here minimal number of quadratic terms in the ODEs had a higher priority than minimal number of reactions. So far it has not been clear whether any at most bimolecular 3-variable system with only four (irreversible) reactions and Hopf bifurcation exists. Meanwhile we found a corresponding system (, , ) which has a supercritical Hopf bifurcation (e.g. at *k*_1 _= *k*_2 _= 1, ). This might be the minimal chemical reaction system with sustained oscillations according to definition (1).

Interestingly, system (2) is similar to a previously presented "minimal reaction network" [12] modelling activation of the epidermal growth factor receptor (EGFR). Our  equation resembles the balance equation of the phosphorylated receptor tyrosine kinase (this superfamily contains EGFR) and  is similar to the differential equation for active protein tyrosine phosphatase.

Bistable systems play important roles also beyond biology. They are usually depicted by the mechanical example of a ball rolling into two different valley basins. Bistable chemical systems, in particular, have been studied extensively to analyse relaxation kinetics [46], non-equilibrium thermodynamics [47], stochastic resonance [48], as well as climate change [49].

Positive feedback is clearly associated with bi/multistability. Negative feedback, in contrast, is often discussed in the context of oscillations [6], and we recently conjectured that this is indeed a necessary condition for (sustained) oscillations [39]. However, this contradicts statements as "sustained oscillations can occur in models based on positive or negative feedback" [50]. Obviously, different understandings of feedback exist. We suggest to use the general Jacobian **J**_**G **_(for system (2) it is given in (3)) for a simple definition of feedback: if the **J**_**G **_terms close any cycles, then feedback exists (e.g. the positive and negative feedback cycles of system (2)), otherwise not. Analysis of **J**_**G **_guarantees a unique identification of all in a system contained feedback cycles. Goldbeter [50] mentioned different examples where the oscillations should be based on a positive feedback, such as glycolytic, Ca^2+^, and cAMP oscillations. However, a more detailed analysis of these systems shows that a negative feedback (according to our definition) is always contained (results unpublished). An example is the simplest model for glycolytic oscillations, the Higgins-Selkov oscillator ([29-31], Table 1): the corresponding general Jacobian is . Its trace and determinant are *v*_2*y *_- *v*_2*x *_- *v*_3*y *_and *v*_2*x*_*v*_3*y*_, respectively. Obviously, *v*_2*y *_in **J**_**G**22 _is the only instability causing term (a positive feedback), so the second reaction is the only ICS in the system. However, inspection of the off-diagonal elements of **J**_**G **_reveals a negative feedback as well: the larger x, the larger becomes y, but the larger y, the smaller becomes x. The same is realised in the bistable system (2), where the positive feedback is the only ICS and another negative feedback is contained. These examples show how the analysis of the general Jacobian helps clarifying the discussion of feedback loops.

We have shown that a mechanism for preventing explosions is a third necessary condition for bistability (complementing the previously discussed two other conditions positive feedback and filtering out of small stimuli). In system (2) this is achieved by a negative feedback. Other bistable systems contain negative feedbacks as well (e.g. ERK pathway [2]), so we hypothesize that this is indeed a typical feature of bistable systems. Interestingly, also oscillating systems typically contain (besides the necessary negative feedback) a positive feedback (for better tunable frequency, evolvability and robustness [51]). Thus, oscillating and bistable systems are practically based on the same set of feedback cycles.

## Conclusion

Bi/multistability and oscillations are the two most important dynamic phenomena in biology. Limit cycle oscillations are associated with biological clocks and cell signalling [52], and spatial oscillations with proper cell division [53]. The fundamental importance of bistability is discussed in the introduction. Some years ago we presented the smallest chemical system with limit cycles [33]. Here we have derived the smallest chemical system with bistability (2).

Minimal systems are well suited for basic studies and for teaching purposes. This explains the great success of, for instance, the Lotka-Volterra [27,28] and the Higgins-Selkov system [30]. We have demonstrated for the minimal chemical reaction system with Hopf bifurcation [33] that it is accessible for detailed mathematic-analytical examination [54] and a good example system for thermodynamic considerations [55]. We hope that also the minimal chemical system with bistability will serve for such purposes in the future.

## Methods

System (2) is the smallest bistable chemical reaction system according to definition (1) - proof (inductive proof systematically considering all possibilities):

1. The Schloegl system (Table 1) is the smallest bistable one-variable (1d) system: 1d systems need an unstable steady state to separate the attractor regions of two stable steady states, so we need at least three steady states to realize a bistable system. The simplest function (which is realizable as a MAK system) f(x) with three zeros is the cubic polynomial. To realize a stable 'on' state the sign of the cubic term needs to be "-" (). For three different non-negative steady states we also need a positive quadratic and a negative linear term: f(x) = -a x^3 ^+b x^2^-c x+d (a, b, c>0) possesses two positive extrema as can simply be seen considering f'(x) = 0. Thus, the minimal bistable 1d system reads . The minimal corresponding MAK system is the Schloegl system shown in Table 1 (a reversible monomolecular efflux reaction (d>0) allows for two positive stable steady states). It follows that a chemically realistic bistable system with only mono- and bimolecular reactions needs at least two variables.

2. Using our general quasi-steady-state-approximation procedure [56], we have previously shown that any irreversible trimolecular reaction can be understood as limit case of a reversible bimolecular reaction and another irreversible bimolecular reaction by introducing one additional intermediate [57]. Transforming the Schloegl system accordingly proves that at most bimolecular bistable 2d systems exist, i.e. the number of variable reactants in the minimal bistable chemical system is fixed to two (cf. definition (1)).

3. The lemma of the index sum [58] states that the sum of indices of all steady states within a two-dimensional confined set (closed region in phase space where all trajectories point inwards [59]) equals one. The index values of a node, a focus, and a saddle are +1, +1, and -1, respectively (for stable and unstable nodes and foci [58]). Our minimal system should therefore contain one unstable (saddle-point) and two stable steady states (we are only considering non-exploding systems, such that a confined set could simply be constructed, trajectories point inward at the boundary of the positive orthant anyway). To get three steady states we need at least a cubic steady state equation f(x) = 0, i.e. an x^3 ^term (higher order polynomials would require more bimolecular reactions). In 2d MAK systems this can only be realized by one ODE with an xy term and the other with x^2 ^and y terms. Inserting the corresponding steady state expression y = x^2^..., into the other ODE's xy term gives the cubic term. A direct x^3 ^term is forbidden in bimolecular systems. The symmetric case y^2 ^and x needs no extra consideration. These terms already correspond to at least three reactions. One can show that such three reactions are not sufficient to give a bistable system (the explicit proof is not necessary, because it turns out indirectly from the following analysis). So the minimal bistable system has at least four reactions.

4. To realize a -x^3 ^term in the corresponding steady state equation, one either needs the terms y and x^2 ^with different signs and -xy in the other ODE, or y and x^2 ^with the same signs and +xy in the other ODE. But the same signs variant cannot work:  is not chemical,  would contain at least one trimolecular reaction,  is not chemical, and  is also impossible. The latter applies because: (i) the  equation must also contain a term -xy (the corresponding reaction cannot be bimolecular otherwise: a term +xy in  implies the bimolecular reaction X+Y->2X, implying the term -xy in ), (ii) detailed analysis of the system  shows that the cubic term in the steady state equation always vanishes, independent of any other added mono- and/or bimolecular reactions. So the y and x^2 ^terms must have different signs in one ODE.

5. There are two variants for the different-sign-case: 1.  2.  (note that the x^2 ^term has to appear also in the  equation to be interpretable as bimolecular reaction). We show that no bistable system with only four reactions exists that corresponds to the first case: to realize a positive quadratic term in the cubic polynomial of the steady state equation (cf. discussion concerning the Schloegl system in 1.)  we have three options: (i) , (ii) , (iii) . The second case could only be realized by 4 different reactions and would need a fifth reaction to yield a linear term in the cubic polynomial (cf. discussion concerning the Schloegl system in 1.). The same holds for the third case, even if another x^2 ^term (still realizable by four reactions) would be added in the  equation. The first case is realizable by three reactions. However, it is easy to see that a linear term in the steady state equation  needs at least two additional reactions.

6. However, the second system  can yield a bistable at most bimolecular system with only 4 reactions: The system  is realizable by three reactions, we add y as the positive term in  (still three reactions) and a fourth reaction, a simple efflux of x to ensure a linear term in the cubic polynomial (to yield three steady states): . Analysis shows that the reaction from y to x needs to be bimolecular (reaction 1 in (2a)) to enable three nonnegative steady states. In fact, this is the only input into the system. Note that this system is quite unique, there is no really different bistable chemical system with only four reactions: the positive term in  has to be y to enable a positive quadratic term in the steady state equation . System (2) is unique with respect to definition (1). The very similar system where reaction 2*X *→ *X *+ *Y *is replaced with 2*X *→ 2*Y *is mathematically equivalent (factor 2 can be incorporated into the kinetic constant). □

The slightly modified system replacing reaction *X *+ *Y *→ *Y *+ *P *with *X *+ *Y *→ *P *is bistable as well (it has also just four reactions, but is not as minimal as system (2) for the third criterion of definition (1). Modifying further by replacing 2*X *→ *X *+ *Y *with 2*X *→ *Y *and *S *+ *Y *→ 2*X *with *S *+ *Y *→ 3*X *gives another bistable system resembling a system which can be derived from the Schloegl-system using our previously discussed general transformation rules [57].
